# High-integrated photonic tensor core utilizing high-dimensional lightwave and microwave multidomain multiplexing

**DOI:** 10.1038/s41377-024-01706-9

**Published:** 2025-01-03

**Authors:** Xiangyan Meng, Nuannuan Shi, Guojie Zhang, Junshen Li, Ye Jin, Shiyou Sun, Yichen Shen, Wei Li, Ninghua Zhu, Ming Li

**Affiliations:** 1 Key Laboratory of Optoelectronic Materials and Devices, Institute of Semiconductors, Chinese Academy of Sciences, Beijing, 100083 China; 2 College of Materials Science and Opto-Electronic Technology, University of Chinese Academy of Sciences, Beijing, 100049 China; 3 School of Electronic, Electrical and Communication Engineering, University of Chinese Academy of Sciences, Beijing, 100049 China; 4 China Academy of Space Technology (Xi’an), Xi’an, Shaanxi 710100 China; 5 WeChat Pay Lab 33, Shenzhen Tencent Computer System Co. Ltd., Shenzhen, 518054 China; 6 Lightelligence Group, Hangzhou, 311121 China

**Keywords:** Microwave photonics, Integrated optics

## Abstract

The burgeoning volume of parameters in artificial neural network models has posed substantial challenges to conventional tensor computing hardware. Benefiting from the available optical multidimensional information entropy, optical intelligent computing is used as an alternative solution to address the emerging challenges of electrical computing. These limitations, in terms of device size and photonic integration scale, have hindered the performance of optical chips. Herein, an ultrahigh computing density optical tensor processing unit (OTPU), which is grounded in an individual microring resonator (MRR), is introduced to respond to these challenges. Through the independent tuning of multiwavelength lasers, the operational capabilities of an MRR are orchestrated, culminating in the formation of an optical tensor core. This design facilitates the execution of tensor convolution operations via the lightwave and microwave multidomain hybrid multiplexing in terms of the time, wavelength, and frequency of microwaves. The experimental results for the MRR-based OTPU show an extraordinary computing density of 34.04 TOPS/mm^2^. Additionally, the achieved accuracy rate in recognizing MNIST handwritten digits was 96.41%. These outcomes signify a significant advancement toward the realization of high-performance optical tensor processing chips.

## Abstract (web summary)

An optical tensor processing unit (OTPU) is proposed, leveraging microwave-lightwave hybrid multiplexing to avoid optoelectronic bandwidth mismatch and employing wavelength-tuning-based weight adjustment to achieve a high computing density of 34.04 TOPS/mm².

## Graphical abstract

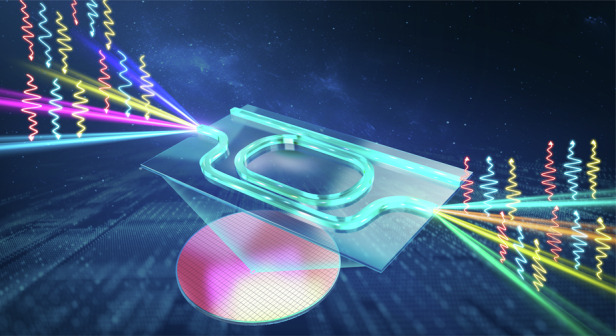

## Introduction

In artificial neural networks (ANNs), the form of data structure is currently classified as vector (one-dimensional array), matrix (two-dimensional array), or tensor (beyond two-dimensional array). Therefore, tensors, as multidimensional arrays embodying specific shapes and data types, play pivotal roles across various domains in contemporary science, such as artificial intelligence (AI)^1–4^, data science^5^, condensed matter physics^6^, and string theory^7^. The intrinsic high-dimensional nature of tensors offers a distinct advantage over classical matrix-based computations, mitigating the risk of multilinear data structure loss. Consequently, tensor computation has evolved into a foundational concept within the realm of ANNs^8,9^, wherein inputs, outputs, and transformations are invariably represented through tensors. The complexity of the data structures and the execution of diverse operations within ANNs are seamlessly managed using tensors. However, the burgeoning landscape of AI-generated content (AIGC) has led to a rapid increase in model parameters. Constrained by the discrete architecture of computing and memory units, the manipulation of extensive tensors exacts a substantial computational cost^10–14^.

To implement high-dimensional tensor calculations, optical computing architectures have emerged as formidable contenders for next-generation computing hardware because of their attributes of ultralarge bandwidth, ultralow power consumption, and ultralow latency^15–19^. Various optical tensor computing chip architectures, such as phase-change materials (PCMs)^20,21^, microring resonators (MRRs)^22^ and Mach–Zehnder interferometers (MZIs)^21^, have been designed utilizing different dimensions and interaction mechanisms of lightwaves to accelerate tensor data processing. In addition to tensor data processing, optical matrix calculations based on wavelength-division multiplexing (WDM)^23,24^, time-division multiplexing^25^, space-division multiplexing^26–29^, and mode-division multiplexing^30^ have also been reported. Despite these advancements, two major challenges remain in optical processing: (1) Most optical computing schemes rely on arrayed units, which limit computing density, and the constrained chip area further restricts integration scale, resulting in lower performance for optical computing chips. (2) The data rate mismatch between high-speed optical computing cores and low-speed electronic processing cores, coupled with the high power consumption of essential high-speed multichannel digital-to-analog and analog-to-digital (DA/AD) converters, impedes the broader commercial adoption of optical computing chips.

To address these challenges, we propose an optical tensor processing unit (OTPU) that leverages a sophisticated fusion of optics and microwave domains, enabling seamless hybrid multiplexing capabilities. Unlike conventional setups reliant on thermo-optic effect MRR-based arrays, our design features an individual thermally tuned-free MRR as the central tensor core. The absolute values of the weights are adjusted by fine-tuning the wavelengths, while the signs (±) of the weights are encoded by switching the intensity modulator (IM) between two quadrature points. By multiplexing multiple physical parameters, including wavelength, time, and microwave frequency—especially with the introduction of microwave frequency—an additional dimension of information expression is introduced. Consequently, the matrix convolution operation is extended to a tensor convolution operation. Following the temporal distribution for data preprocessing technology, the ultrahigh-integration architecture of MRR-based OTPU enables a remarkable computing density of 34.04 TOPS/mm^2^. Handwritten digit recognition from the MNIST database^31^ achieved an accuracy of 96.41%, which is consistent with the theoretical expectation of 96.79%. This innovative optical tensor convolution architecture, employing a single MRR as the basic optical computing unit, substantially reduces the chip footprint by N^2^-1 times and facilitates the high computing power realization of optical computing chip in limited size. The introduction of microwave-subcarriers improves the data input channels which enables tensor operation and improves flexibility of information access in end-side application scenarios, such as biometric feature recognition and telemedicine. Furthermore, the multimodal computing paradigm that combines lightwave and microwave technologies transforms the processing of high-speed data into a multi-channel, low-speed data processing framework. This innovative approach mitigates the reliance on high-speed DA/AD converters, paving the way for a significant leap in the practical application of optical computing.

## Results

### Principle

**Fig. 1:**
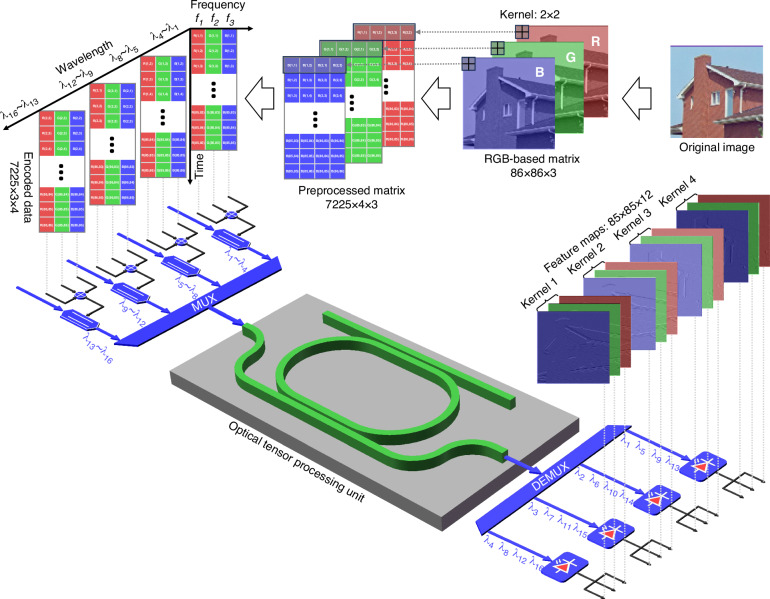
**The principle of optoelectronic integrated tensor convolution processing utilizing the proposed OTPU**. MUX multiplexing, DEMUX demultiplexing

The optoelectrical multidomain hybrid tensor convolution process is illustrated in Fig. 1. Our proposed architecture is composed of two main processes for different functional needs: 1) wavelength/frequency division multiplexing and 2) optical tensor convolution. In the first process, the input data are preprocessed to complete the mapping of digital tensor convolutions to microwave and lightwave multimodal convolutions. In the other process, optical tensor convolution processing is accomplished with the proposed OTPU, and the final feature maps are output from the optical chip.

**Fig. 2:**
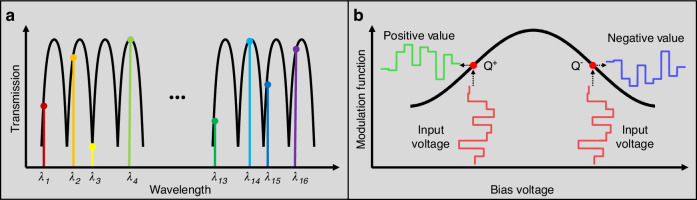
**The principle of real-valued tensor kernel realization. a** Absolute value adjustment of weights based on single passive MRR through wavelength tuning. **b** Modulation function of IM. The positive and negative signs of the weights are achieved by setting the bias voltage of the IM at the Q^+^ and Q^−^ points

In the proposed OTPU, the MRR is employed as the core component to implement tensor convolution, where the desired energy is absorbed depending on the relative position between the working wavelength and the resonance wavelength of the MRR. When the wavelength is in the on-resonance state, almost all the energy of the light is entrapped in the MRR, and the weight is labeled “0”. Similarly, the wavelength is adjusted to fix it in the off-resonance state, and nearly no light energy is stored in the MRR; thus, the weight value is labeled “1”. Owing to the ability of WDM technology to individually tune the wavelength of light carriers, different elements in the tensor kernel are loaded to the intensity of different light carriers through a single passive MRR one by one. Figure 2a shows the implementation of weighting by wavelength adjustment based on a single MRR, where the difference weights between “0” and “1” are individually loaded one by one by wavelength tuning.

Electronic integrated circuits manipulate electrical signals using a transistor, capacitance, inductance and resistance to support digital computations. In contrast, photonic integrated circuits are more suitable for analog information processing and handle lightwaves using lasers, modulators, optical processing devices and photodetectors. In the analog computing paradigm, the role of an optical processing device is equivalent to that of transistors in digital circuits. In this paper, the MRR serves as an optical processing device to independently execute tensor convolution. Similar to its role in electronic integrated circuits, an individual MRR, as the perfect functional processing unit, forms the cornerstone of a photonic tensor core referred to as the OTPU.

Another important consideration in Fig. 1 is the preprocessing of image data and the subsequent encoding of the matrix into the individual wavelength of the laser array. Conceptually, from spectrum slicing for microwave photonic channelized technology, tensor calculations are executed through the introducing of microwave-subcarriers. Herein, the parallel tensor encoding method is applied to handle the input image matrix. As illustrated in Fig. 1, an RGB-based image matrix with three dimensions, height, width and channel, is mapped to three information entropies in the microwave photonics of wavelength, time and space. To implement the tensor convolution operation, the original image is divided into three monocolour matrices, named the R-based matrix, G-based matrix and B-based matrix, and then converted into three color channel matrices with 7225 rows and 4 columns, where the number of rows corresponds to the number of kernel slides and the number of columns is equal to the number of elements in the kernel. Subsequently, each color channel matrix (R, G, B) is converted into four sequential vectors by column. Then, four vectors from the same color channel matrix are modulated on four groups of light carriers with the same microwave-subcarrier signal, and three microwave-subcarriers are used for three color channels. The detailed data preprocessing and encoding steps used for image matrix recombination are described in Supplementary Note 1.

After data preprocessing and loading the matrices to optical carriers by microwave frequency multiplexing combined with wavelength division multiplexing, the tensor convolution operation is accomplished in the OTPU. The tensor convolution of an RGB-based colorful image with four 2 × 2 kernels is illustrated in Fig. 1, where one tensor convolution operation is essentially matrix–matrix multiplication between a 4 × 4 matrix $W$ and a 4 × 3 matrix $X$. One row in matrix $W$ represents one kernel, and one column in matrix $X$ represents one encoded data channel. The calculation result of one tensor convolution is expressed as

1 $$\left[\begin{array}{ccc}{y}_{R1} & {y}_{G1} & {y}_{B1}\\ {y}_{R2} & {y}_{G2} & {y}_{B2}\\ {y}_{R3} & {y}_{G3} & {y}_{B3}\\ {y}_{R4} & {y}_{G4} & {y}_{B4}\end{array}\right]=\left[\begin{array}{c}\begin{array}{cccc}{w}_{11} & {w}_{12} & {w}_{13} & {w}_{14}\end{array}\\ \begin{array}{cccc}{w}_{21} & {w}_{22} & {w}_{23} & {w}_{24}\end{array}\\ \begin{array}{cccc}{w}_{31} & {w}_{32} & {w}_{33} & {w}_{34}\end{array}\\ \begin{array}{cccc}{w}_{41} & {w}_{42} & {w}_{43} & {w}_{44}\end{array}\end{array}\right]\left[\begin{array}{ccc}{x}_{R1} & {x}_{G1} & {x}_{B1}\\ {x}_{R2} & {x}_{G2} & {x}_{B2}\\ {x}_{R3} & {x}_{G3} & {x}_{B3}\\ {x}_{R4} & {x}_{G4} & {x}_{B4}\end{array}\right]$$

where $y$ represents the convolution results, $w$ represents the elements in the kernels and $x$ represents the input data values. In tensor convolution, sixteen light carriers (${\lambda }_{1}-{\lambda }_{16}$) are selected to represent sixteen elements (${w}_{11}-{w}_{44}$) in four 2 × 2 kernels, and three microwave-subcarriers ${f}_{1}-{f}_{3}$ are utilized to represent three matrix channels (${x}_{R}$, ${x}_{G}$ and ${x}_{B}$). As shown in Fig. 1, the sixteen light carriers are divided into four groups, and one kernel is represented by four wavelengths from the four groups respectively. For instance, the first kernel (${w}_{11}-{w}_{14}$) is represented by the wavelengths ${\lambda }_{1}$, ${\lambda }_{5}$, ${\lambda }_{9}$ and ${\lambda }_{13}$. All the elements in matrix $W$ are weighted through an MRR-based tensor core, and each element can be individually adjusted by tuning the corresponding wavelength of the laser array. Benefiting from hybrid multiplexing of both the microwave-based frequency and light-based wavelength in our work, a single MRR cell rather than the MRR array independently implements high-dimensional parallelism computation. Compared to the WDM-based computing paradigm, the introduction of microwave frequency is more conducive to meeting the computing requirements of side-to-side arrangements for multiple inputs/outputs.

Furthermore, the indisputable fact remains that optical intensity is unattainable for the representation of negative value weights. Therefore, researchers have made critical efforts to find ways to solve negative weighting problems, such as optical-field subtraction between the signal light and the reference light^17,18,24,32^ and electronic-field phase-differential preprocessing^33^. As shown in Fig. 2b, the bias voltage of the Mach–Zehnder modulator (MZM) in the modulation function is switched between two quadrature points (${Q}^{+}$ and ${Q}^{-}$) to realize both positive and negative kernel elements within the optical domain. Specifically, the quadrature points of ${Q}^{+}$ with a positive modulation slope were set to $-{V}_{\pi }/2$, and the remaining quadrature points of ${Q}^{-}$ with a negative modulation slope were set to ${V}_{\pi }/2$ (${V}_{\pi }$, the half-wave voltage, which is the direct voltage used to generate the $\pi$ phase shift of the modulator). If the direct voltage is biased at $-{V}_{\pi }/2$, the output signal has an identical phase relationship with the input signal; if the direct voltage is switched at ${V}_{\pi }/2$, the output signal is the inverse of the input signal. Thus, positive and negative weight values are achieved by setting the bias voltage between $-{V}_{\pi }/2$ and ${V}_{\pi }/2$, avoiding the use of balance photodetector^32^ or the unwanted electrical subtraction after the photodetector array^17,18^.

### Experimental setup and optical tensor processing unit

**Fig. 3:**
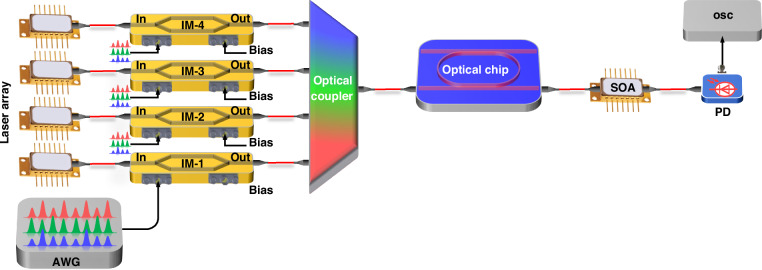
**Diagram of the experimental structure of optoelectrical tensor convolution processing.** IM intensity modulator, SOA semiconductor optical amplifier, PD photodetector, OSC oscilloscope, AWG arbitrary waveform generator

To execute the matrix‒matrix multiplication shown in Eq. (1), the preprocessed vector is encoded in the amplitude of the microwave-subcarrier and subsequently loaded on the amplitude of wavelengths one by one, thus enabling multiple calculations simultaneously. Figure 3 shows a sketch of the high-dimensional parallel tensor convolution operation for a sheet of a colorful image. Three preprocessed vectors are generated and grouped together into an encoded matrix and output from one channel of an arbitrary waveform generator using frequency division multiplexing in the microwave field, and four analog signals are output from four channels of the arbitrary waveform generator (AWG) (details about data encoding can be found in Supplementary Note 2). Then, the encoded matrix is loaded on four continuous waves of light from the laser array using an electro-optical IM. After combining the wavelengths with an optical coupler, multiplication is carried out in the MRR-based cell. A semiconductor optical amplifier (SOA) is inserted to compensate the optical loss behind the MRR. Subsequently, the addition operation is accomplished with a photodetector (PD). The convolution results are recorded in a high-speed oscilloscope. Finally, the stored data are divided into three electrical channels, down-converted and filtered to show the feature maps with a digital computer (details about microwave demultiplexing and down-conversion can be found in Supplementary Note 3).

**Fig. 4:**
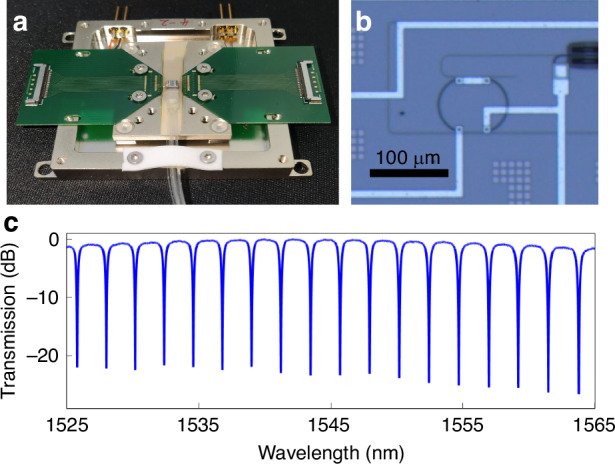
**The optical tensor convolution processing chip. a** Image depicting the packaged optical tensor convolution processing chip. **b** Microscopic view of the optical tensor convolution processing chip. **c** Transmission spectrum characteristics of the optical tensor convolution processing chip

The MRR as a main cell for implementing tensor computation was fabricated in a commercial SiPh foundary on a 200 mm wafer running with a standard 90 nm lithography process. Figure 4a shows a photograph of the packaged optical tensor convolution processing chip. To maintain a stable working state, a thermoelectric cooler (TEC) is installed at the bottom of the chip to detect and control the chip temperature. The optical tensor convolution processing chip is coupled with the fiber array using edge couplers, and the measured coupling loss for one edge coupler is ~1.50 dB. The MRR was fabricated via a standard silicon-on-insulator integration process (Fig. 4b). The footprint for a single MRR is ~7207.50 μm^2^ with a length of 93.00 μm, width of 77.50 μm, and insert loss less than 0.10 dB. Figure 4c shows the measured transmission response result of the designed MRR, revealing a 2.24 nm channel spacing and 1.51 dB channel flatness within the FSR. It provides the normalized weights of every FSR with the variation in the applied wavelength (methods for weight adjustment and calibration can be found in Supplementary Note 4).

### Optical tensor convolution

**Fig. 5:**
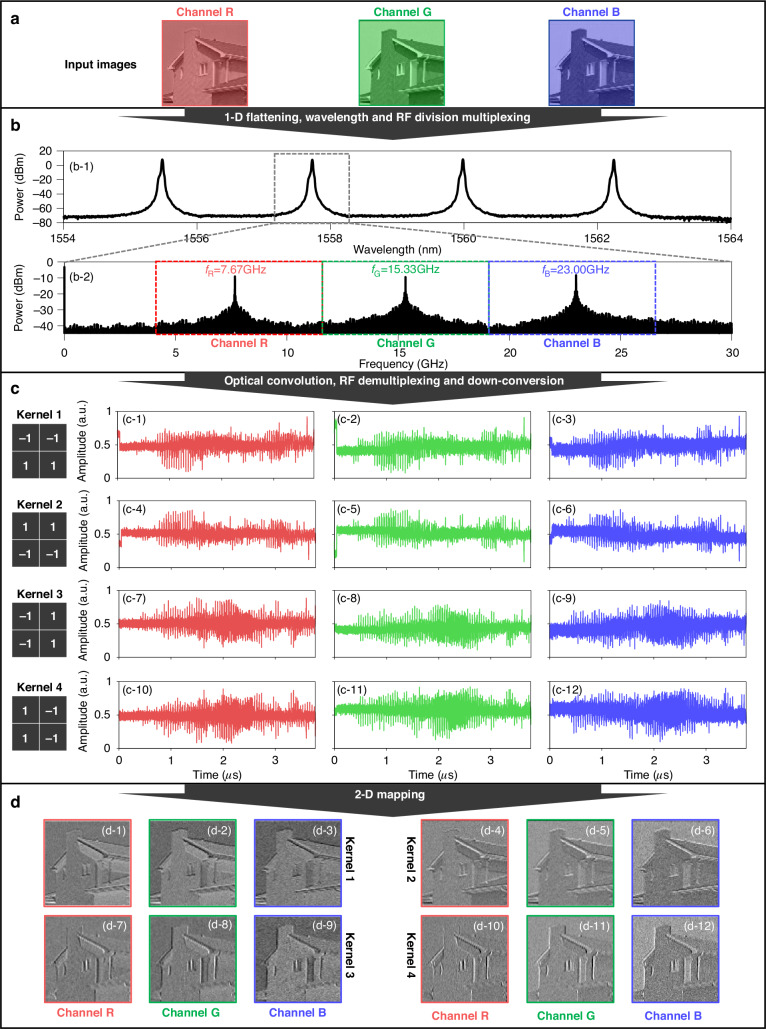
**Experimental results of optoelectrical tensor convolution processing. a** Input images comprising three channels. **b** The wavelengths utilized for computation and the microwave spectrum of the input data to one IM. **c** Waveforms post optical convolution, microwave demultiplexing, and down-conversion. **d** The resultant feature maps

In the optical tensor convolution experiment, an RGB-based image from the USC-SIPI image database is down-sampled and decomposed into three monochrome image matrices with a size of 86 × 86 for each image, as shown in Fig. 5a. Four real-valued 2 × 2 kernels, $$\left[\begin{array}{ll}-1 & -1\\ 1 & 1\end{array}\right]$$, $$\left[\begin{array}{ll}1 & 1\\ -1 & -1\end{array}\right]$$, $$\left[\begin{array}{ll}-1 & 1\\ -1 & 1\end{array}\right]$$ and $$\left[\begin{array}{ll}1 & -1\\ 1 & -1\end{array}\right]$$, are formed for tensor feature extraction demonstration in four experiments. The reconfiguration of real-valued kernels is realized by wavelength adjustment of lasers and work point adjustment of IMs, where the absolute value reconfiguration is finished by wavelength adjustment and sign reconfiguration is accomplished by working point tuning of the IM. Therefore, four wavelengths set at 1555.48 nm, 1557.74 nm, 1559.97 nm and 1562.25 nm are chosen to ensure that all working wavelengths are within the flattop of the transmission response in the MRR to realize the absolute value of “1”. The bias voltage of the MZM is used for transforming the signs of the kernel weights by switching between $-{V}_{\pi }/2$ and ${V}_{\pi }/2$. The frequencies of these three chosen microwave-subcarriers are 7.67 GHz (channel R), 15.33 GHz (channel G) and 23.00 GHz (channel B), for a data rate of 1.92 GBaud. Figures 5b1–2 show the spectrograms of the weighted wavelengths with a 0.02 nm resolution and the microwave spectrum of the encoded signal modulated at a wavelength of 1557.74 nm.

After computing in the OTPU, the output waveform is divided into three channels and mixed with three local oscillator signals centered at 7.67 GHz, 15.33 GHz and 23.00 GHz to down-convert the output signal. Then, these down-converted signals are filtered with lowpass filters to filter out the expected computing results, and the corresponding waveforms are shown in Fig. 5c. Figures 5c1–3 show the three-channel down-conversion signals for kernel $$\left[\begin{array}{ll}-1 & -1\\ 1 & 1\end{array}\right]$$, which are the average of 5 acquisitions in one experiment to reduce noise; the other three kernels are also shown in Fig. 5c4–12. The rearranged 2-D feature maps recovering from the temporal waveform from Fig. 5c are shown in Fig. 5d, where Fig. 5d-1) corresponds to Figs. 5c-1) and the others are analogous. Details about the influence of different microwave-subcarriers on computing precision can be found in in Supplementary Note 5.

### Digit classification with a CNN

**Fig. 6:**
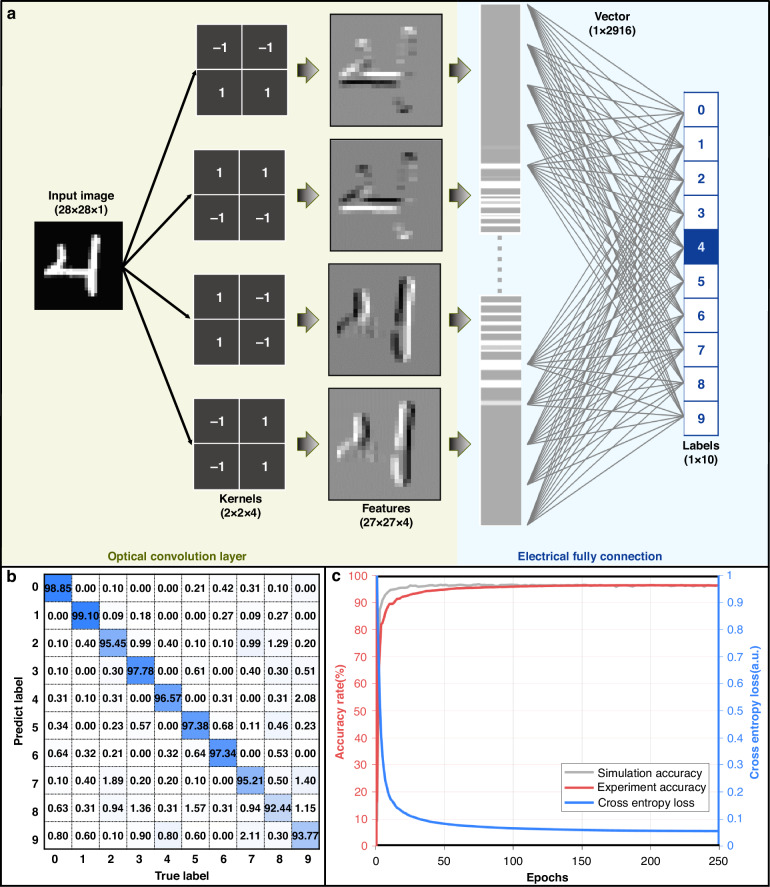
**Results of handwritten digit recognition. a** Architecture of the CNN utilized in the experimental setup for handwritten digit recognition. **b** Confusion matrix depicting the recognition results for 10,000 images from the test dataset of the MNIST database. **c** Fluctuations in simulation accuracy, experimental accuracy, and cross-entropy loss over the course of 250 training iterations, which yields a final experimental recognition accuracy of 96.41%

Based on wavelength-dependent weight adjustment methods, the optical convolution chip is used as the optical convolutional layer. After the convolution operation in the optical domain, the feature maps are nonlinearly activated and fully connected with a digital computer, and an opto-electrical convolutional neural network is established for handwritten digit recognition. As shown in Fig. 6a, in the optical convolutional layer, four 2 × 2 kernels are utilized in four experiments to extract the edge features of the top, bottom, right and left. For each experiment, the 28 × 28 input image is first flattened into a 729 × 4 data matrix, and four columns of the data matrix are loaded to four optical carriers through four IMs with a data rate of 30.67 GBaud. After the convolution operation in the optical domain, the feature maps are nonlinearly activated with the ReLU function and fully connected in the electrical domain, and a 1 × 10 vector is output. In the output vector, the index of the maximum value indicates the recognition result.

In the experiment, handwritten digits from the MNIST database were utilized for recognition demonstration. A total of 60,000 images from the training dataset were utilized for training, and 10,000 images from the test dataset were used for testing. After 250 epochs of training, the experimental recognition accuracy rate reached 96.41%, which is consistent with the theoretical accuracy rate of 96.79%. Figure 6b shows the confusion matrix, where the abscissa indicates the true label and the ordinate shows the predicted label. Figure 6c shows the changes in accuracy and cross-entropy loss during the training process, where the gray line and red line show the simulation and experimental accuracy, respectively, and the blue line shows the experimental cross-entropy loss.

When performing the convolution operation of the 2 × 2 kernel, eight operations are completed in one period. With a data rate of 30.67 GBaud, the computing speed of the chip is 30.67 GBuad × 8 = 245.33 GOPS, and the computing density of the optical computing chip reaches 245.33 GOPS ÷ 7207.50 μm^2^ = 34.04 TOPS/mm^2^. Table 1 presents a performance comparison of representative computing frameworks, including the MZI^34^, MRR^35,36^, PCM^20^ and multimode interference (MMI)^24^. The proposed architecture shows superiority in terms of recognition accuracy, wavelength occupation, data rate and especially experimental computing density. Compared to other general optical computing architectures^20,36^, the experimental computing density shows an order of magnitude improvement, which provides a new path to overcome the performance bottleneck caused by the scale of optical integrated chips. In addition, as a result of the single MRR architecture, both the computational speed and the computing density can be easily improved by introducing additional wavelengths to improve parallelism and increase the data rate.

**Table 1:** Performance comparison of the proposed OTPU

Type	Platform	Accuracy on MNIST test set	Network architecture	Wavelengths	Data rate (GBaud)	Efficiency (/OPS)	Precision of results	Experimental compute density (TOPS/mm^2^)
MZIs^34^	Si	76.70% (4 categories, vowel recognition)	2 FC (O)	1	/	15.00 fJ^a^	5-bit	1.12^a^
MRRs^35^	Si	97.41%	3 FC (O)	4	10	0.2 pJ^b^	4-bit	5.78^b^
MRRs^36^	Si	96.60%	1 Conv. (O) + 2 FC (E)	4	17.00	0.42 pJ^a^	/	1.04
PCMs^20^	SiN	95.30%	1 Conv. (O) + 1 FC (E)	36	2.00	2.5 pJ	7-bit	0.20
MMI^24^	SiN	92.17%	1 Conv. (O) + 1 FC (E)	4	16.60	2.42 pJ	5-bit	25.48^c^
Nvidia H100	Si	/	/	/	1.40	0.35 pJ	8-bit	2.43
This work	Si	96.41%	1 Conv. (O) + 1 FC (E)	4	30.67	3.75 pJ	5-bit	34.04

“O” represents computing in optical domain and “E” represents computing in electrical domain

^a^These data can be obtained based on existing state-of-the-art equipment

^b^Data derived from a large-scale outlook of the proposed structure

^c^The rows in the matrix are correlated with each other

## Discussion

The optical tensor core accelerates graphic processing through the multilevel coupling mode of MRRs, specifically utilizing an MRR-based array. Traditionally, these methods rely on the thermo-optical effect to adjust the refractive index of MRRs for weight assignment, necessitating the configuration of an MRR array to establish a weight matrix iteratively. However, integrating thermo-optical effects to regulate MRRs within compact chips often induces significant thermal crosstalk, posing challenges in independently fine-tuning each MRR. To alleviate the impact of thermal crosstalk in the MRR array, maintaining specific distances between different MRRs is customary, resulting in an expanded chip area. Techniques such as substrate hollowing are employed to minimize heat conduction between waveguides, thereby increasing chip processing complexity. Additionally, intricate control circuits and sophisticated feedback algorithms are essential for addressing the issue of weight mismatch. In the proposed OTPU architecture, a single thermally tuned-free MRR serves as the pivotal component for optical tensor processing. The overall temperature control of the chip is achieved solely using a TEC, ensuring stable chip operation. This approach eliminates the thermal crosstalk challenges associated with utilizing thermo-optical effects for weight control, thereby simplifying both the chip production process and control complexity. Moreover, the absence of thermal crosstalk between MRRs allows for a more compact arrangement, leading to a higher computing density.

Furthermore, the advantages of the large bandwidth and low power consumption inherent in photonic signal processing are pivotal for the superior performance of optical computing chips^15,37^. However, current optical computing chips, which serve as dedicated accelerators, require collaboration with microelectronic chips to achieve comprehensive computing functionalities. Consequently, rapid conversion between analog and digital signals becomes imperative. Typically, the substantial power consumption associated with this high-speed DA/AD conversion severely constrains the exploitation and widespread adoption of optical computing chip advantages^20,22,32,36,38^. The proposed OTPU adopts the microwave-subcarrier approach^21^. This approach not only facilitates tensor operations based on a single MRR but also circumvents the need for high-speed DA/AD converters, effectively mitigating the power consumption inherent in such conversion processes. Due to the limitations of our lab conditions, microwave multiplexing (details about hardware implementation can be found in Supplementary Note 2) and demultiplexing (details about hardware implementation can be found in Supplementary Note 3) in the experiment were conducted digitally using a computer. In practical applications, these processes including microwave multiplexing and demultiplexing can be conducted using single-frequency microwave sources, low-speed DA/AD converters (≤5 GSa/s), and passive electrical mixers, combiners, splitters, and filters. This strategic maneuver alleviates a critical bottleneck, thereby advancing the practical deployment of optical computing chips.

Additionally, enhancing the overall computing power is the pursuit of excellence for optical tensor processors and it is essential to discuss the scalability of the proposed OTPU. Augmenting the number of wavelengths utilized within each MRR is the most intuitive way to enhance the parallelism of computations. By increasing the number of wavelengths in each MRR, both the matrix size and optical power are enhanced due to the use of more lasers. This facilitates the development of high-performance optical computing chips and mitigates the power issues encountered in other passive arrays when scaling up. The development of on-chip tunable lasers^39^ as well as high-resolution wavemeters^40^ also addresses the demands for precise wavelength adjustment in the OTPU (details about integrated tunable lasers and wavemeters can be found in Supplementary Note 6). On the other hand, analogous to conventional microelectronic chips, a convenient method for calling up the chip’s computing power is to duplicate the MRR-based OTPU on a large scale to form a multicore parallel computing paradigm (details about scalability of OTPU can be found in Supplementary Note 7).

In summary, a novel optical tensor convolution processing chip designed on a silicon platform is introduced in this paper, offering a streamlined approach to tensor computational tasks. Central to the design is a singular MRR serving as the fundamental unit for optical computation. By exploiting the transmission spectrum characteristics of the MRR, wavelengths are dynamically adjusted to correspond with various resonance states, thereby tuning the absolute values of the kernels. Real-valued kernels are derived through the manipulation of operating points of IMs. The tensor convolution operation is facilitated by the introduction of microwave subcarriers, which effectively augment the dimensionality of the input data. In the experimental validation, optical tensor convolutions are performed on a color image featuring three channels (R, G, and B) utilizing 2 × 2 kernels where three microwave subcarriers and four wavelengths are occupied. Additionally, handwritten digit recognition tasks utilizing the MNIST database were conducted, achieving a data rate of 30.67 GBaud and an accuracy rate of 96.41%, closely aligning with the theoretical projections of 96.79%. Notably, the architecture of the basic optical computing unit is compact, with a groundbreaking computing density record of 34.04 TOPS/mm^2^. Owing to the absence of active reconfiguration requirements for optical chips, optical chips theoretically operate with zero power consumption. Furthermore, the strategic utilization of microwave-subcarriers leverages the expansive bandwidth of optical computing, reducing data rates and eliminating the necessity for power-intensive high-speed DA/AD converters. This approach effectively addresses a significant obstacle, thus facilitating the practical application of optical computing technologies.

## Materials and methods

### Configuration

Optical tensor convolution computing with the proposed OTPU was implemented using commercially available optoelectronic components. The laser array is an IDPHOTONICS CoBrite-DX laser source with four tunable polarization-maintaining output ports to generate four wavelengths, where the wavelength tuning resolution is 1 MHz. An arbitrary waveform generator (AWG) from Keysight is used to generate a four-channel analog modulation signal with a sample rate of 92 GSa/s (M8196A). Four electro-optical intensity modulators from iXblue (MX-LN-40) follow the laser array to load the image data to the intensity of the lightwave carriers. One polarization-maintaining 1 × 4 optical coupler from THORLABS (TPQ1315HA) is utilized to combine the four wavelengths into one optical path. After computing in the OTPU, a semiconductor optical amplifier from THORLABS (SOA1013S) is inserted into the computing link to compensate the optical loss. Then, the optical signal is converted to an electrical signal with a Finisar photodetector (XPDV2150R), which has a bandwidth of 50 GHz. Finally, a high-speed oscilloscope from Keysight (UXR0402A) with a sample rate of 256 GSa/s was utilized for data recording.

## Supplementary information

Supplementary Information

## Data Availability

The data that support the findings of this study are available from the corresponding authors upon request.
