# Supplementary material for: High-integrated photonic tensor core utilizing high-dimensional lightwave and microwave multidomain multiplexing
Source: Light Sci Appl. 2025 Jan 3;14:27. doi: 10.1038/s41377-024-01706-9 (PMC11697043; doi:10.1038/s41377-024-01706-9)
Supplement: Supplementary file 1 — Supplementary Information [file 41377_2024_1706_MOESM1_ESM.pdf]

Supplementary Information for

High-integrated photonic tensor core utilizing high-dimensional lightwave and microwave multidomain multiplexing

Authors:

Xiangyan Meng[1,2,3], Nuannuan Shi[1,2,3*], Guojie Zhang[4], Junshen Li[1,2,3], Ye Jin[1,2,3], Shiyou Sun[5], Yichen Shen[6], Wei Li[1,2,3], Ninghua Zhu[1,2,3] and Ming Li[1,2,3*]

Affiliations:

[1] Key Laboratory of Optoelectronic Materials and Devices, Institute of Semiconductors, Chinese Academy of Sciences, Beijing 100083, China

[2] College of Materials Science and Opto-Electronic Technology, University of Chinese Academy of Sciences, Beijing 100049, China

[3] School of Electronic, Electrical and Communication Engineering, University of Chinese Academy of Sciences, Beijing 100049, China

[4] China Academy of Space Technology (Xi'an), Xi'an, Shaanxi, 710100 China

[5] WeChat Pay Lab 33, Shenzhen Tencent Computer System Co., Ltd. Shenzhen 518054, China

[6] Lightelligence Group, Hangzhou 311121, China

*Corresponding authors: nnshi@semi.ac.cn and ml@semi.ac.cn

Table of Contents

Supplementary Note 1: Methods of data preprocessing and encoding 2
Supplementary Note 2: Data encoding methods used in the experiment 3
Supplementary Note 3: Microwave de-multiplexing and down-conversion 5
Supplementary Note 4: Weight value calibration 6
Supplementary Note 5: The bit precision of OTPU 6
Supplementary Note 6: On-chip tunable lasers and high-resolution wavemeter for the OTPU 8
Supplementary Note 7: Scalability of the proposed OTPU 9
Supplementary Note 8: Energy efficiency and compute density 11
Supplementary Note 9: Weight adjustment resolution of the proposed OTPU 13
Supplementary Note 10: The influence of microwave-subcarrier on computing capability 14

Supplementary Note 1: Methods of data preprocessing and encoding

The process of flattening the RGB image data is shown in Fig. S1, which uses tensor convolution between the RGB-based color image and four 2×2 kernels as an example. The original image, which contains three color channels, $\begin{bmatrix} R_{1,1} & R_{1,2} & & \\ R_{2,1} & R_{2,2} & & \\ & & \ddots & \\ & & & R_{86,86} \end{bmatrix}$, $\begin{bmatrix} G_{1,1} & G_{1,2} & & \\ G_{2,1} & G_{2,2} & & \\ & & \ddots & \\ & & & G_{86,86} \end{bmatrix}$ and $\begin{bmatrix} B_{1,1} & B_{1,2} & & \\ B_{2,1} & B_{2,2} & & \\ & & \ddots & \\ & & & B_{86,86} \end{bmatrix}$, is first flattened into sequential vectors. Using channel R of the color image as an example, the 86×86 image is divided into four 85×85 sub-images $\begin{bmatrix} R_{1,1} & R_{1,2} & & \\ R_{2,1} & R_{2,2} & & \\ & & \ddots & \\ & & & R_{85,85} \end{bmatrix}$, $\begin{bmatrix} R_{1,2} & R_{1,3} & & \\ R_{2,2} & R_{2,3} & & \\ & & \ddots & \\ & & & R_{85,86} \end{bmatrix}$, $\begin{bmatrix} R_{2,1} & R_{2,2} & & \\ R_{3,1} & R_{3,2} & & \\ & & \ddots & \\ & & & R_{86,85} \end{bmatrix}$ and $\begin{bmatrix} R_{2,2} & R_{2,3} & & \\ R_{3,2} & R_{3,3} & & \\ & & \ddots & \\ & & & R_{86,86} \end{bmatrix}$. Then, each sub-image is flattened by row into a 7225×1 vector, and a 7225×4 matrix of $\begin{bmatrix} R_{1,1} & R_{1,2} & R_{2,1} & R_{2,2} \\ R_{1,2} & R_{1,3} & R_{2,2} & R_{2,3} \\ R_{1,3} & R_{1,4} & R_{2,3} & R_{2,4} \\ \vdots & \vdots & \vdots & \vdots \\ R_{85,83} & R_{85,84} & R_{86,83} & R_{86,84} \\ R_{85,84} & R_{85,85} & R_{86,84} & R_{86,85} \\ R_{85,85} & R_{85,86} & R_{86,85} & R_{86,86} \end{bmatrix}$ is generated for one color image channel. For the three color image channels, three 7225×4 preprocessed matrices can be obtained.

After flattening, three preprocessed 7225×4 matrices are recombined to accomplish the process of microwave frequency multiplexing and wavelength division multiplexing.

Each 7225×4 matrix is multiplexed into one microwave-subcarrier (the R channel for f_1, the G channel for f_2 and the B channel for f_3), and four columns in each 7225×4 matrix are multiplexed into four wavelength groups (the first column for $\lambda_1-\lambda_4$, the second column for $\lambda_5-\lambda_8$, the third column for $\lambda_9-\lambda_{12}$ and the last column for $\lambda_{13}-\lambda_{16}$). After wavelength and microwave multiplexing, the encoded 7225×3×4 data are obtained and fed to the optical tensor processing unit (OTPU). The data fed to the OTPU in one period can be expressed as

$$X=\begin{bmatrix} x_{R1} & x_{G1} & x_{B1} \\ x_{R2} & x_{G2} & x_{B2} \\ x_{R3} & x_{G3} & x_{B3} \\ x_{R4} & x_{G4} & x_{B4} \end{bmatrix} \tag{1}$$

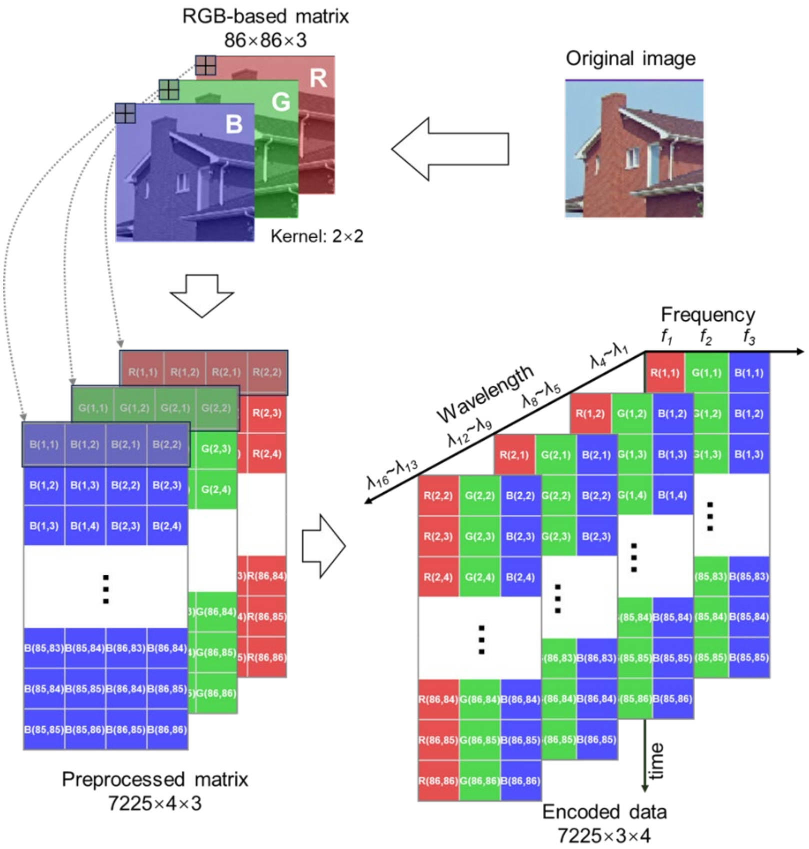

Fig. S1. Principle of RGB data preprocessing.

Supplementary Note 2: Data encoding methods used in the experiment

In the experiment, due to the limitations of our lab's experimental conditions, microwave division multiplexing is implemented digitally using a computer rather than with analog hardware. In the process of microwave division multiplexing, for 2×2 kernels, four inputs with continuous-time data representation were mathematically generated in Python 3.7, and converted to .csv files readable by the arbitrary waveform generator (AWG). Four analog modulation signals are generated with an AWG to modulate four groups of lightwave carriers through four intensity modulators.

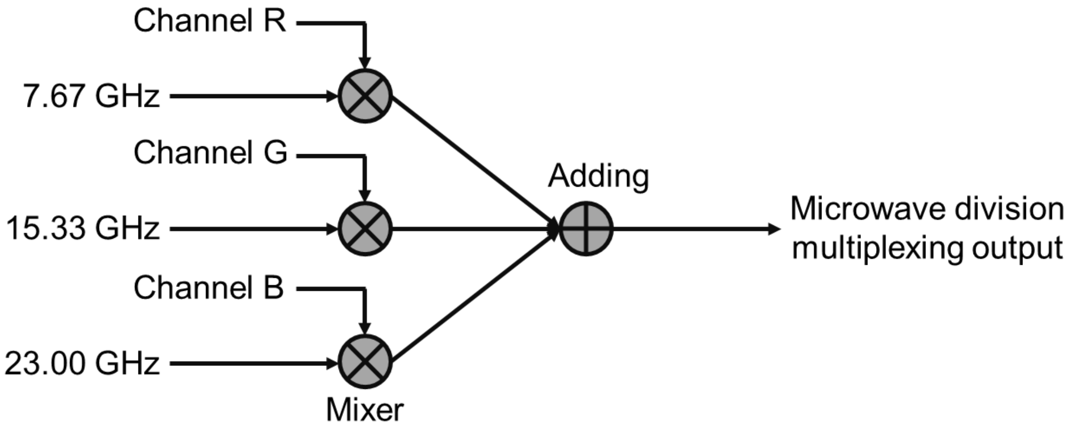

Fig. S2. The encoding method of data input to one of the AWG channels.

Fig. S2 shows the encoding method of data input to one of the AWG channels. For each analog modulation signal, three microwave signals with frequencies of 7.67 GHz, 15.33 GHz and 23.00 GHz are first generated as the microwave-subcarriers. Since the sample rate of the arbitrary waveform generator is set to 92 GSa/s and the data rate is set to 1.92 GBaud, each data point is sampled 48 times, and three 7225×1 vectors from three preprocessed matrices are first upsampled into 346800×1 vectors. Then, three 346800×1 vectors are multiplied with three microwave-subcarriers (three 346800×1 vectors) to complete the up-conversion process, and three up-converted 346800×1 vectors are

obtained. After up-conversion, three up-converted vectors are combined into one 346800×1 vector by adding three up-converted vectors and fed to the AWG to convert to one channel of the analog modulation signal.

Based on the structure shown in Fig. S2, data encoding can be easily achieved using electrical hardware including single-frequency microwave sources, low-speed data generators (≤5 GSa/s), and passive electrical mixers and combiners. This approach eliminates the need for high-speed, high-power data generators.

Supplementary Note 3: Microwave de-multiplexing and down-conversion

After processing with the proposed optical tensor processing unit, the output signal is recorded by the oscilloscope and transmitted to the computer via local area network (LAN). The process of microwave division de-multiplexing was also mathematically implemented in Python 3.7. The principle of microwave de-multiplexing and down-conversion for one output channel is illustrated in Fig. S3.

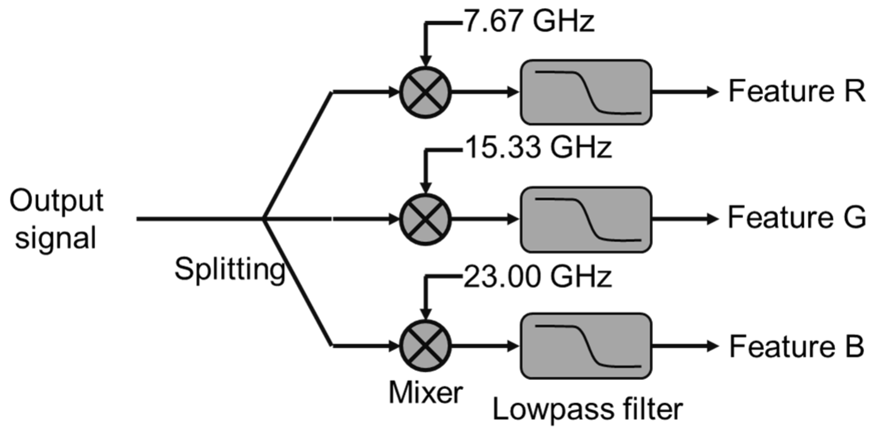

Fig. S3. Principle of microwave de-multiplexing and down-conversion of one output channel.

The computing result is split into three microwave-subcarrier channels and mixed with three local oscillator signals centered at 7.67 GHz, 15.33 GHz and 23.00 GHz to down-

convert the output signal. After down-conversion, the computing result for each microwave-subcarrier channel is transferred to the DC carrier. Finally, three down-converted signals are filtered with three lowpass filters with a pass band of DC-1.92 GHz to filter out the expected computing results, and three features are obtained.

Similar to Supplementary Note 2, the process of microwave de-multiplexing and down-conversion is implemented digitally. Based on the structure shown in Fig. S3, data de-multiplexing and down-conversion can be easily achieved using electrical hardware, including low-speed data recorders (≤5 GSa/s), single-frequency microwave sources, passive electrical splitters, mixers and filters, thereby avoids the use of high-speed, high-power data recorders.

Supplementary Note 4: Weight value calibration

To mitigate the impact of slight variations in light amplitude across different FSRs in the MRR, the relative intensities of various wavelengths are used for weight encoding. During the experiment, these weights are adjusted by monitoring the relative intensities with an Optical Spectrum Analyzer. Amplitude fluctuations among adjacent FSRs are generally minor. To further reduce the effect of these fluctuations, adjacent wavelengths are selected to represent the same kernel. Additionally, comparisons of relative intensities are only made between wavelengths corresponding to the same kernel, while those representing different kernels are compared separately.

Supplementary Note 5: The bit precision of OTPU

The bit precision N_b [1] is defined by the following equation:

$$N_b = \log_2\left(\frac{\mu_{max} - \mu_{min}}{\sigma}\right) \quad (2)$$

where μ_{max} and μ_{min} denote the upper and lower bounds of the output values, respectively, while σ represents the standard deviation of discrepancies between the experimental and theoretical outputs.

To calculate the bit precision of the proposed OTPU, a calibration process is conducted using the convolution results from 60,000 images in the MNIST training dataset, which are processed with four different kernels. This amounts to a total of 27×27×4×60000=174.96 million operations. According to the calculation, the standard deviation of $\sigma = 0.0411$ is obtained, which is shown in Fig. S4a. The calculation results are normalized to 1 so that $\mu_{max} = 1$ and $\mu_{min} = 0$, thereby determining the bit precision to be equivalent to 4.6-bit (The integers are rounded to 5-bit in Table 1 of the main text.).

To analyze the effect of microwave-subcarriers on computing bit precision, the bit precisions of different microwave-subcarriers are calculated individually. The computing results of four kernels are used to calibrate the computing precision for three microwave-subcarriers, as shown in Fig. 5 of the manuscript. For each microwave-subcarrier, 85×85×4=28900 operations are performed for the calibration. The standard deviations for the three microwave-subcarriers are calculated as 0.0377, 0.0456, and 0.0564, corresponding to microwave-subcarrier frequencies of 7.67 GHz, 15.33 GHz, and 23.00 GHz, respectively. The error distribution histograms are shown in Fig. S4b-d respectively. These values correspond to bit precisions of 4.7-bit, 4.4-bit, and 4.1-bit. The errors for different microwave-subcarriers are less than 1-bit, which is acceptable for neural networks.

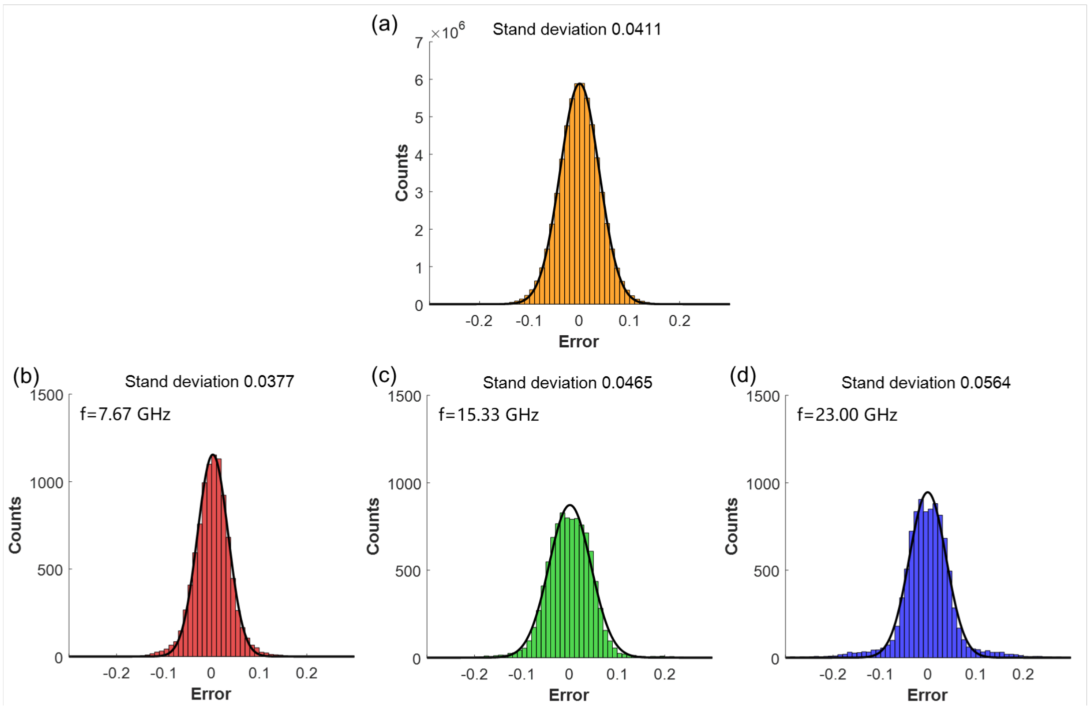

Fig. S4. Error distribution histograms. (a-d) show the error distributions without a microwave subcarrier and with microwave subcarrier frequencies of 7.67 GHz, 15.33 GHz, and 23.00 GHz, respectively.

Supplementary Note 6: On-chip tunable lasers and high-resolution wavemeter for the OTPU

For the proposed OTPU, which is primarily designed for inference tasks, a very high weight update rate is not required, and the demand for wavelength tuning speed is relatively low. The main requirements for photonic integrated circuit (PIC)-based tunable lasers are wavelength accuracy and laser linewidth. Meanwhile, on-chip high resolution wavemeter is another important device for fine wavelength tuning.

Currently, commonly used PIC-based tunable lasers include distributed feedback (DFB) lasers, distributed Bragg reflector (DBR) lasers, external cavity lasers, and vertical cavity surface emitting lasers (VCSELs). Among these lasers, external cavity lasers show larger wavelength tuning range, higher optical output power, narrower laser linewidth and easier to control. Considering the application requirements of OTPU, along with

advantages of external cavity laser make it the most suitable PIC-based tunable laser for OTPU. The external cavity laser is a relatively mature technology, capable of achieving narrow linewidths (ranging from tens to hundreds of kHz) with a wavelength tuning range of more than 40 nm[2-4]. Additionally, on-chip integration of external cavity lasers is straightforward, accomplished through coupling with semiconductor optical amplifiers and on-chip mode-selective external cavities.

In addition to tunable lasers, precise control of the wavelength is crucial for the proposed OTPU, making high-resolution on-chip wavelength monitoring (wavemeter) indispensable. Various on-chip wavemeter solutions have been extensively reported[5-7], including structures like arrayed waveguide gratings, MZIs, MZIs couples with multimode interference[5]. These approaches have demonstrated high-resolution wavelength monitoring capabilities. On-chip wavemeters with a broad wavelength range of 80 nm[6] and high-resolution wavemeters achieving resolutions on the order of MHz[7] have been reported, fulfilling the requirements for OTPU applications.

Supplementary Note 7: Scalability of the proposed OTPU

Scalability is a crucial aspect of the proposed OTPU architecture. In this design, matrix scalability is primarily achieved by increasing the number of wavelengths. Each matrix element corresponds to a specific tunable wavelength, so the first method for expanding the OTPU is to increase its optical bandwidth. By optimizing the design of components such as the MRR, the system can support multiple optical bandwidths simultaneously, thereby increasing the number of available wavelengths.

The second method to scale the OTPU is by adding more FSRs within the operating optical bandwidth, taking into account the maximum frequency of the microwave subcarrier. The most straightforward approach is to increase the ring length of the MRR, which reduces the FSR and allows more FSRs to fit within the optical bandwidth. Additionally, using a material with a higher refractive index to fabricate the MRR is another way to reduce the FSR.

However, the optical bandwidth cannot be infinite, nor can the FSR be infinitely small. Therefore, the number of wavelengths that an MRR can support is always limited. To build larger matrices, a passive MRR array, which is not cascaded among MRRs, can be utilized, as illustrated in Fig. S5.

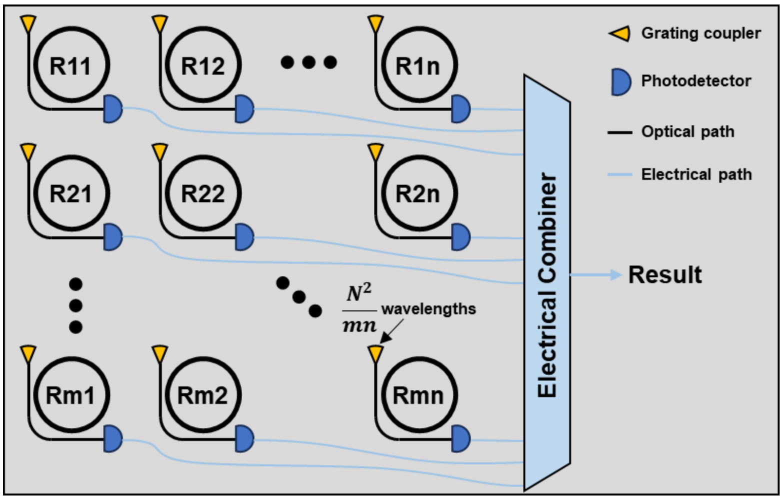

Fig. S5. Scaling Method of the proposed OTPU.

When using an m×n MRR array to implement an N×N computational matrix, each MRR handles $N^2/(m \times n)$ tunable wavelengths. Together, the m×n MRRs manage the weight control for all N^2 elements in the matrix. The output from each MRR is converted to an electrical signal by a photodetector, and these signals are then summed using an electrical combiner to obtain the final calculation result. $N^2/(m \times n)$ tunable wavelengths are needed

to located in $N^2/(m \times n)$ FSR of each MRR, and MRRs in the passive MRR array operating in the same optical band. Therefore, tunable optical wavelengths in different MRRs may be the same depending on the weights in the matrix and transmission curve of different MRRs. By this way, the large-scale computing is implemented, which avoids the challenges of large optical power loss and error accumulation in passive cascaded arrays, such as MZIs and MRRs.

With the MRR shown in Fig. 4, utilizing the entire C band for computation allows for 18 FSRs, accommodating up to 18 wavelengths. For instance, using this MRR to perform a 64×64 matrix operation, which represents the state-of-the-art in on-chip optical computing, the matrix can be divided into 256 4×4 submatrices. This would require 256 MRRs, each processing 16 wavelengths. These 256 MRRs can be fabricated in a commercial foundry, demonstrating the potential for large-scale expansion of the proposed OTPU architecture.

Supplementary Note 8: Energy efficiency and compute density

The energy required for lasers to counteract shot noise and the capacitance of photodetectors, assuming a constant bit precision N_b, is expressed by the following formula[8]:

$$P_{lasers} \geq \frac{hv}{\eta} \max(2^{2N_b+1}, \frac{CV_d}{e}) f \tag{3}$$

Here, h is the Planck constant, v is the optical frequency corresponding to a central wavelength of 1550 nm, $C = 2.4$ fF and $V_d = 1$ V are the capacitance and driving voltage of the photodetectors[9], η is the combined quantum efficiency of the laser, photodetector, and optical link loss ($\eta \approx 31$ dB, assuming a 10% quantum efficiency of the laser and

photodetector), e is the elementary charge, f is the data rate (30.67 Gbaud/s in the experiment). The overall energy consumption of the computing system is described by another equation:

$$P_{total} = P_{lasers} + NP_{DAC} + NE_{mod}N_b f + P_{ADC} \tag{4}$$

Here, N is the number of channels, which is set to 4 in this scenario. P_{DAC}, E_{mod}, N_{ps} and P_{ADC} correspond to the energy consumption of the digital-to-analog converter (DAC), modulator, phase shifter, and analog-to-digital converter (ADC), respectively. The figure of merit (FoM) for DAC is given by $FoM = 2^B \bullet f_s / P_{DAC}$, where B is the bit precision of DAC, f_s is the sample rate, and P_{DAC} is the energy consumption of DAC[10]. Referring to a reported DAC with 14-bit precision, a 10 GS/s sampling rate, and an energy consumption of 177 mW[11], a DAC with the same FoM, 8-bit precision, and a 30.67 GS/s sampling rate would have an energy consumption of approximately 8.48 mW. The energy consumption for the ADC and modulators are estimated to be $P_{ADC} = 199$ mW[12] and $E_{mod} \approx 1$ pJ/b[13], respectively.

Thus, given the parameters of N_b=5 and f=30.67 Gbaud/s, the total power consumption is calculated to be 0.92 W following Eq. (4). The maximum computing speed is calculated as 245.33 giga-operations per second (GOPS). Consequently, the efficiency is 3.75 pJ/OPS.

In terms of computing density, when performing convolution operations with a 2×2 kernel, 8 operations are completed within one period. With a data rate of 30.67 GBaud, the chip achieves a computing speed of 30.67 GBaud × 8 = 245.33 GOPS, resulting in a computing density of 245.33 GOPS ÷ 7207.50 μm^2 = 34.04 TOPS/mm^2 [14-21]. A

performance comparison of representative computing frameworks is provided in Table 1 of the main text.

Supplementary Note 9: Weight adjustment resolution of the proposed OTPU

Weight resolution is important for matrix computing accuracy, and we calculate it theoretically through the gradient of the transmission curve of the MRR. According to Fig. 4(c), the gradient of the transmission curve of the MRR is shown in Fig. S6. The maximum gradient of the transmission curve of the MRR is -480.20 dB/nm, resulting in the maximum optical power fluctuation of 0.0038 dB with the wavelength tuning resolution of 1 MHz (IDPHOTONICS CoBrite-DX laser source, 100 GHz corresponding to about 0.8 nm in C band). According to Eq. (2), by using the maximum fluctuation of optical power instead of the standard deviation (which is a stricter measure since the standard deviation is generally smaller than the maximum error), the weight precision theoretically reaches 10-bit accuracy. In the experiment, due to the influence of optical power fluctuations and noise in the link, the weight accuracy will be reduced, but the MRR-based weight control is still the current record holder for the accuracy of optical computing weight control (9-bit)[18,22].

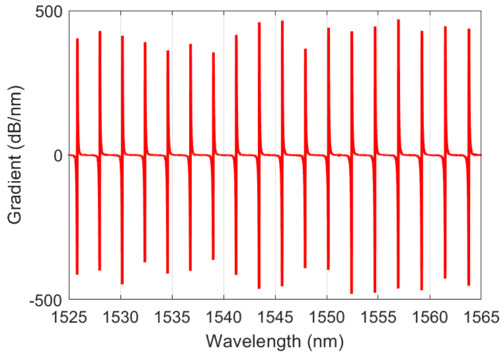

Fig. S6. The gradient of the transmission curve of the MRR.

In addition, microwave subcarriers are employed in the experiment. The wavelength tuning resolution will uniformly affect all microwave subcarriers, as each wavelength carrier

serves as the common carrier for all microwave subcarriers within the same data input channel.

As for the influence of wavelength stability, given that the wavelength tuning resolution is 1 MHz (from the datasheet of laser source), the maximum wavelength instability is significantly less than 1 MHz, causing a much smaller impact compared to wavelength adjustment errors.

Supplementary Note 10: The influence of microwave-subcarrier on computing capability

Theoretically, using frequency division multiplexing reduces computing capability by 50% for the same optical bandwidth, since a double sideband modulation signal with a bandwidth of m GHz occupies 2m GHz of bandwidth. Despite this reduction in computing capability, it supports tensor computing and eliminates the need for high-frequency data generators and recorders, which is highly beneficial for practical optical computing processes.

To address the problem of reduced computing capability caused by microwave subcarriers, single sideband modulation — an approach widely used in microwave photonics—can be employed. With single sideband modulation, a signal with a bandwidth of m GHz occupies only m GHz of communication bandwidth, thus avoiding the reduction in computing power associated with microwave subcarriers.

1 Tait, A. N. *et al.* Feedback control for microring weight banks. *Opt. Express* **26**, 26422-26443 (2018).
2 Komljenovic, T. *et al.* Widely tunable narrow-linewidth monolithically integrated external-cavity semiconductor lasers. *IEEE J. Sel. Top. Quantum Electron.* **21**, 214-222 (2015).
3 Ken, S., Tomohiro, K. & Hirohito, Y. in *Proc.SPIE.* 79431G (2011).
4 Youwen, F. *et al.* in *Proc.SPIE.* 91351B (2014).

5 Yao, X. S. Sine-cosine optical frequency detection devices for photonics integrated circuits and applications in lidar and other distributed optical sensing. United States patent 11 619 783 (2023).
6 Stern, B., Kim, K., Gariah, H. & Bitauld, D. Athermal silicon photonic wavemeter for broadband and high-accuracy wavelength measurements. *Opt. Express* **29**, 29946-29959 (2021).
7 Xiang, C. *et al.* Integrated chip-scale si3n4 wavemeter with narrow free spectral range and high stability. *Opt. Lett.* **41**, 3309-3312 (2016).
8 Nahmias, M. A. *et al.* Photonic multiply-accumulate operations for neural networks. *IEEE J. Sel. Top. Quantum Electron.* **26**, 1-18, 7701518 (2020).
9 Chen, L. & Lipson, M. Ultra-low capacitance and high speed germanium photodetectors on silicon. *Opt. Express* **17**, 7901-7906 (2009).
10 *An electro-photonic system for accelerating deep neural networks* (2021).
11 Huang, H. Y., Chen, X. Y. & Kuo, T. H. A 10-gs/s nrz/mixing dac with switching-glitch compensation achieving sfdr >64/50 dbc over the first/second nyquist zone. *IEEE J. Solid-State Circuits* **56**, 3145-3156 (2021).
12 Kull, L. *et al.* in *2018 IEEE Symposium on VLSI Circuits.* 275-276 (2018).
13 Miller, D. A. B. Device requirements for optical interconnects to silicon chips. *Proc. IEEE* **97**, 1166-1185 (2009).
14 Zhou, H. *et al.* Photonic matrix multiplication lights up photonic accelerator and beyond. *Light Sci. Appl.* **11**, 30 (2022).
15 Ashtiani, F., Geers, A. J. & Aflatouni, F. An on-chip photonic deep neural network for image classification. *Nature* **606**, 501-506 (2022).
16 Feldmann, J. *et al.* Parallel convolutional processing using an integrated photonic tensor core. *Nature* **589**, 52-58 (2021).
17 Xu, X. *et al.* 11 tops photonic convolutional accelerator for optical neural networks. *Nature* **589**, 44-51 (2021).
18 Bai, B. *et al.* Microcomb-based integrated photonic processing unit. *Nat. Commun.* **14**, 66 (2023).
19 Cheng, J. *et al.* Multimodal deep learning using on-chip diffractive optics with in situ training capability. *Nat. Commun.* **15**, 6189 (2024).
20 Meng, X. *et al.* Compact optical convolution processing unit based on multimode interference. *Nat. Commun.* **14**, 3000 (2023).
21 Xu, Z. *et al.* Large-scale photonic chiplet taichi empowers 160-tops/w artificial general intelligence. *Science* **384**, 202-209 (2024).
22 Zhang, W. *et al.* Silicon microring synapses enable photonic deep learning beyond 9-bit precision. *Optica* **9**, 579-584 (2022).
